# A Cluster of Three snoRNAs Including Jouvence Required in the Gut Determines Lifespan and Confers Neuroprotection Through Metabolic Parameters

**DOI:** 10.1111/acel.70464

**Published:** 2026-03-31

**Authors:** Sara Al Issa, Théo Gauvrit, Patricia Daira, Nathalie Bernoud‐Hubac, Jean‐René Martin

**Affiliations:** ^1^ Équipe: Imagerie Cérébrale Fonctionnelle et Comportements (ICFC) Institut des Neurosciences Paris‐Saclay (Neuro‐PSI) UMR‐9197, CNRS/Université Paris‐Saclay Saclay France; ^2^ INSA Lyon, CNRS, LaMCoS, UMR5259, 69621 Villeurbanne France

**Keywords:** aging, cholesterol, gut/brain axis, longevity, metabolism, neurodegeneration/neuroprotection, snoRNA, triglycerides

## Abstract

In our society, the aging of the population is a major public health concern. Recently we have identified a new snoRNA (jouvence) in Drosophila, and showed that its deletion (F4) reduces lifespan, while its overexpression increases it. F4 deleted flies also present neurodegenerative lesions and a deregulation of metabolic parameters such as triglycerides and sterols. However, a deeper characterization of this genomic locus has revealed the presence of two additional snoRNAs. Here, we have characterized, at the whole‐organism level, the role of each of them. First, we show that each snoRNA is expressed in the epithelium of the gut (in the enterocytes), and in the fat body. Second, in the context of the F4 deletion, the re‐expression of each snoRNA in the enterocytes or in the fat body is sufficient to improve lifespan and protect against neurodegeneration in old flies. In addition, according to snoRNA, it rescues the expression of specific deregulated genes within the epithelium of the gut that are involved in lipid and sterol metabolism. Consequently, these two metabolic parameters are also rescued, establishing a relationship between each snoRNA and the lesions of the brain, the metabolic disorders, and the lifespan. Finally, histological stainings revealed that the neurodegenerative lesions are due to an increase of free sterol within the brain and lipid peroxidation in the pericerebral fat body. These results point to a causal relationship between the snoRNAs' function in the epithelium of the gut and the neurodegenerative lesions through the metabolic parameters, revealing a gut‐brain axis.

## Graphical abstract

The disruption of a cluster of three snoRNAs in the epithelium of the gut deregulates the lipid and sterol (including the cholesterol) homeostasis. At the whole‐organism level, this chronic deregulation, all along life, decreases lifespan and leads to neurodegenerative lesions in old flies.

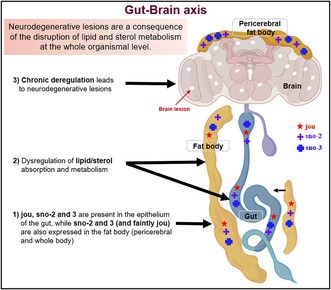

## 1 Introduction

In our society, aging, longevity, and metabolic disorders are major concerns of public health. They result from complex biological processes of accumulation of damage at molecular, cellular, tissue, organ, and whole organism levels (Singh et al. 2019; Fontana et al. 2010). This high complexity is related to the fact that neurodegenerative diseases are progressive disorders with typically late‐onset increase, making it difficult to precisely decipher the genetic and molecular origins of their causes, in other words, where and when they occurred. Genetic, molecular, and physiological approaches have revealed several genes and signaling pathways involved in the determination of aging, lifespan, and neurodegeneration (López‐Otín et al. 2013, 2016, 2023; Gems and Partridge 2013; Giannakou and Partridge 2007; Broughton et al. 2005; Owusu‐Ansah and Perrimon 2014; Biglou et al. 2021). Up to now, these multiple causes have been grouped into 12 hallmarks of aging (López‐Otín et al. 2013, 2023), reflecting their diversity. Among them, the insulin signaling pathway has been the most studied (Giannakou and Partridge 2007; Broughton et al. 2005; Biglou et al. 2021). Moreover, several studies have emphasized the role of lipid metabolism (Johnson and Stolzing 2019; Liu et al. 2015).

In addition, other metabolic parameters, such as sterol (including cholesterol) homeostasis have also been implicated in aging and neurodegeneration, both in Drosophila (Jing and Behmer 2020; Tschäpe et al. 2002) and in mammals (Saher 2023). For example, the NPC1b gene promotes sterol absorption from the midgut epithelium (Voght et al. 2007), while the NPC1a causes cholesterol aggregation and age‐progressive neurodegeneration (Phillips et al. 2008). The NPC type C‐2 gene family controls sterol homeostasis and steroid biosynthesis, and has been used as a model of human neurodegenerative diseases (Huang et al. 2007). Drosophila cannot synthesize sterols, and thus are dependent on the various forms of sterol contained in the diet (Carvalho et al. 2010). More recently, the microRNAs have also been shown to be major contributors in aging and neurodegeneration (Eacker et al. 2009; Liu et al. 2012; Kato and Slack 2013; Abe and Bonini 2013; Soulé et al. 2020; Soulé and Martin 2020).

Here, we have characterized the role of a new cluster of three snoRNAs including *jouvence*, in the control of longevity, neurodegeneration and their relationship to lipids and sterol metabolism. First, we show that each snoRNA is expressed in the epithelium of the gut, and to a lesser extent in abdominal and pericerebral fat bodies. Second, in the F4‐deleted flies, in which the entire cluster is removed, the re‐expression of each snoRNA, individually, or in combinations of two or three, either in the epithelium of the gut or in the fat body is sufficient to increase to various degrees the lifespan, and to protect against neurodegeneration. At the physiological level, the snoRNAs and their combinations also differently rescue the metabolic parameters, such as triglycerides and sterol levels and composition. These rescued metabolic parameters are also supported by the restored expression of some specific genes, previously shown to be deregulated within the epithelium of the gut of F4‐deletion flies (Soulé et al. 2020), that are involved in lipids and/or sterol metabolism and associated with each snoRNA. Finally, histological stains (Nile Red and BODIPY C11‐581/591) of the brains indicate that the neurodegenerative lesions are due, at least in part, to complex perturbation of sterol homeostasis within the brain, and lipid peroxidation in the pericerebral fat body. Since the snoRNAs are not expressed within the brain, but are required and sufficient in the epithelium of the gut (and to a lesser extent within the fat body) to prevent the development of neurodegenerative lesions, these results highlight a causal relationship between the epithelium of the gut and the neurodegenerative lesions linked by metabolic parameters, implying a gut‐brain axis.

## 2 Results

### 2.1 The [PGal4]4C Locus Contains a Small Cluster of 3 snoRNAs

**FIGURE 1:**
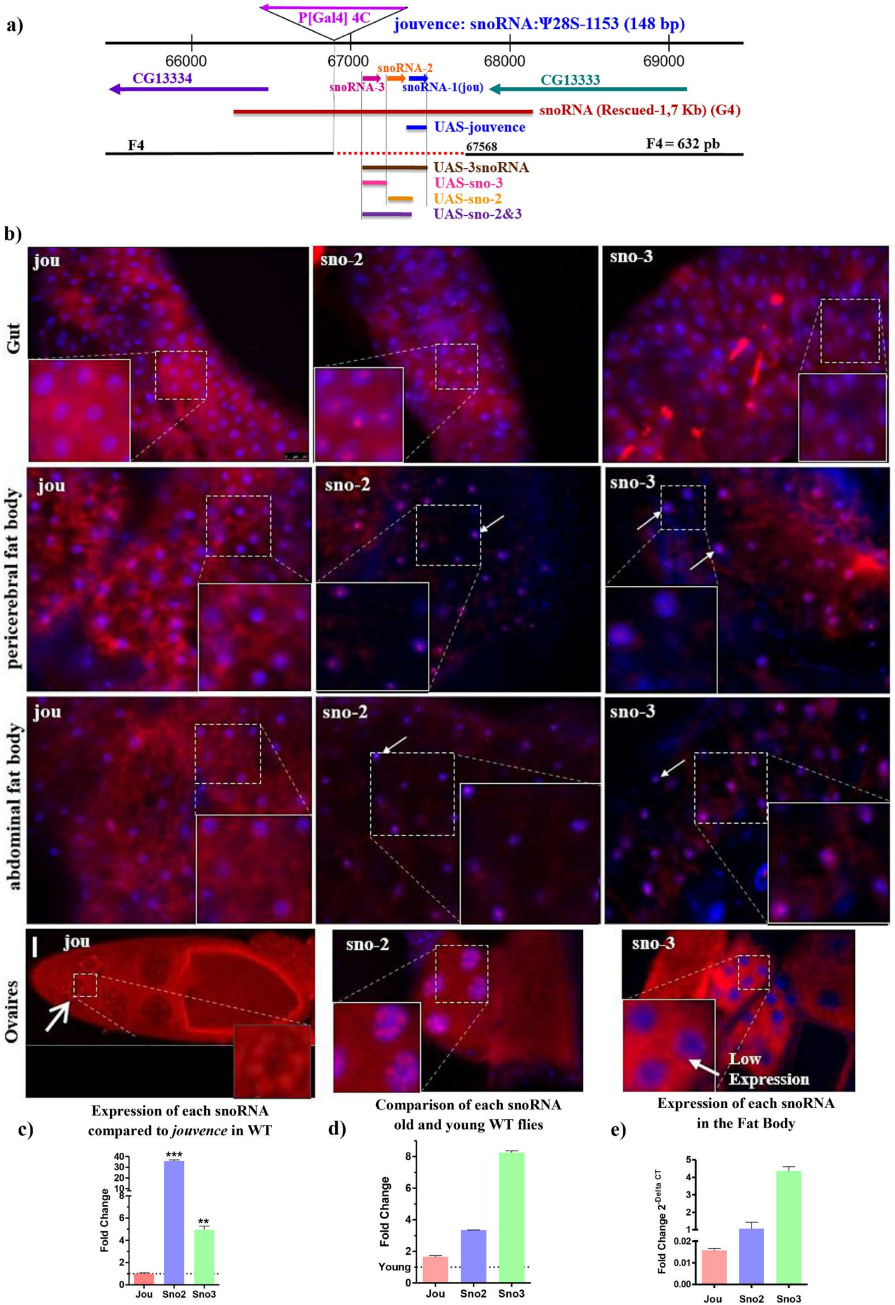
Molecular map and the expression pattern of the 3 snoRNAs. (a) The snoRNA:Ψ28S‐1153 (jouvence, corresponding to the sno1) and the two other snoRNAs (sno‐2 and sno‐3) are in the inverse orientation of the two protein‐coding genes: CG13333 and CG13334. The deletion (F4) of 632 bp (red dotted line) encompasses the 3 snoRNAs. The 1723 bp genomic DNA fragment (red bar) (G4) used for a rescuing transgene. The snoRNA fragment (blue bar) of 148 bp used to generate UAS‐jou construct, the 166 bp fragment (orange bar) used for UAS‐sno‐2, the 157 bp fragment (pink bar) used for UAS‐sno‐3, the 356 bp fragment (purple bar) used for UAS‐sno‐2&3, and the 517 bp fragment (brown bar) used for UAS‐3 snoRNA. (b) In situ Hybridization (ISH) of each snoRNA, jou, sno‐2 and sno‐3 on whole fly cryostat sections reveals a restricted expression (in red, white arrows) in the epithelium of the gut, in the pericerebral and the abdominal fat bodies. Each of them are also expressed in the nurse cells of the ovary (for jouvence, see also Soulé et al. 2020). Nuclear DAPI staining is in blue. Scale bar = 25 μm. Insets (magnification) showing that each snoRNA are located in the nucleolus. (c, d) RT‐qPCR (Taqman) showing the expression level of each snoRNAs on dissected gut of Wild‐Type CS flies. (c) In young (7 day‐old), the snoRNA levels are normalized to the level of rp49. The expression level of jou was used as a reference and set to 1. (d) In 40 day‐old flies, the snoRNA levels are normalized to the level of rp49 and are presented relative to the levels observed in 7‐day‐old flies, used as the reference (set to 1, represented by dotted line on the histogram). (e) in fat body, RT‐qPCR (Taqman) showing the expression level (fold change—delta CT) calculated to the level of rp49, of each snoRNAs on dissected abdominal fat body. Three independent biological replicates were performed (*n* = 3). Statistics: Compared to jouvence level used as reference = 1. (*p*‐values) (*p < 0.05; **p < 0.005; ***p < 0.0005). Errors bars represent the mean ± SEM (*p*‐value were calculated using the student's *t*‐test, using Prism).

Recently, we have identified a new small nucleolar RNA (snoRNA) (*jouvence*) in Drosophila, and showed that its deletion reduces lifespan, while its overexpression increases it (Soulé et al. 2020). To achieve this function, *jouvence* is required and sufficient in the epithelium of the gut. However, more recently, a developmental transcriptomic analysis has annotated two additional snoRNAs (snoRNA:2R:9445205, here named sno2, and snoRNA:2R:9445410, here named sno3) (Graveley et al. 2011) localized just upstream to the first snoRNA:Ψ28S‐1153 (*jouvence*, which can be considered as the sno1). Molecular genetics confirmed the presence of these two snoRNAs, revealing a small cluster of three snoRNAs (Figure 1a). sno2 and sno3 share a high degree of homology (Soulé et al. 2020) and match the canonical definition of the H/ACA box snoRNAs (Kiss 2002; Ye 2007) since they form a hairpin‐hinge‐hairpin‐tail structure (Figure S1). Bioinformatic analysis predicts that they do not pseudouridylate the same ribosomal sites as jouvence, which remains to be determined experimentally (Figure S12 for the putative 2D‐structure and putative pseudouridylation sites for the sno2 and sno3). These three closely located snoRNAs are separated by only ~50 bp, and all are included within the F4‐deletion. Therefore, we wished to determine their individual roles, as well as their putative functional relationships. Each one of them is expressed in the enterocytes of the epithelium of the gut, as well as in the ovary, and to a lesser extent in the fat body (Figure 1b). However, their expression levels and patterns slightly differ. As previously shown, *jouvence* is expressed in all enterocytes of the midgut (Soulé et al. 2020), while sno2 and sno3 are also expressed in the midgut, but not in all enterocytes (Figure 1b). The sno2 and sno3 are also expressed in some cells of the abdominal and pericerebral fat bodies, where *jouvence* is only faintly, and not conclusively detectable, by ISH in this tissue. RT‐qPCR confirms the expression of each snoRNA, as well as their relative levels of expression, both in enterocytes (Figure 1c,d), and in the fat bodies (Figure 1e). In brief, in the gut and in the fat bodies, sno2 and sno3 are more highly expressed than *jouvence*, both in young and old flies.

### 2.2 Targeted Expression of Each snoRNA Increases Lifespan

**FIGURE 2:**
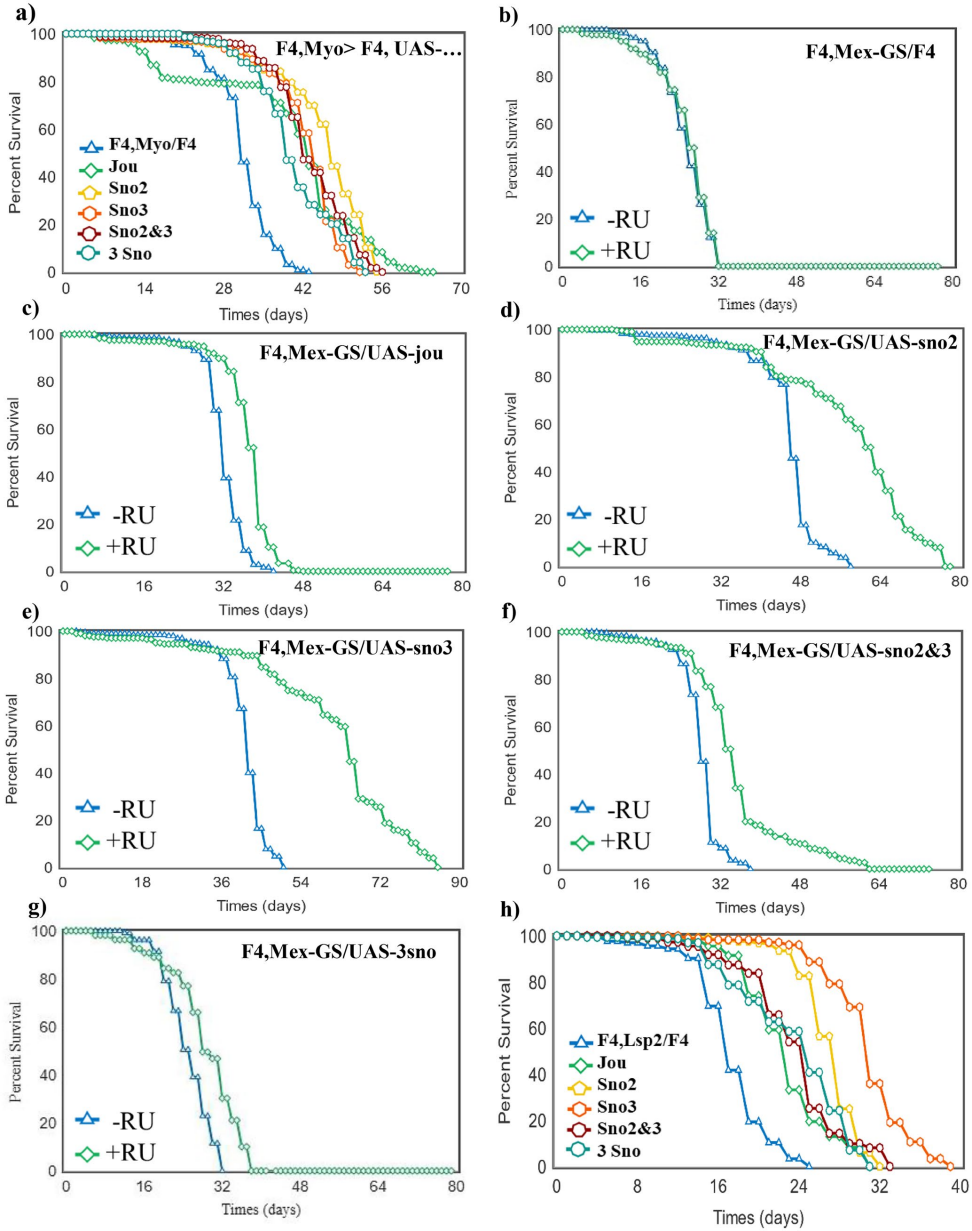
Targetted expression of each snoRNA in the enterocytes or in the fat body increases lifespan. Longevity test results (survival curve—decreasing cumulative) of the targeted expression of each snoRNA specifically in the enterocytes or in the fat body in F4 flies compared to the F4 flies that do not express any snoRNA. (a) Myo1A‐Gal4 driving each UAS‐snoRNA in the enterocytes. (b) F4, Mex‐GS/F4 fed with RU486. (c–g) Mex‐GS driving each UAS‐snoRNA in enterocytes fed with RU486. (h) Lsp2‐Gal4 driving each UAS‐snoRNA in the abdominal and pericerebral fat bodies. For the number of flies, age in days at % mortality, and detailed statistics, see Table S2. *p*‐value calculated by log‐rank test using OASIS Sofware.

In order to determine which snoRNAs, individually or in combination, are required to rescue longevity in F4‐deletion genetic background, we used three P[Gal4] drivers: Myo1A‐Gal4 and two inducible Gene‐Switch (Mex‐GS and CG8997‐GS) (see Figure S1 for their expression pattern) whose activity can be induced only in adulthood by feeding the flies with Mifepristone (RU486). When individually expressed specifically in the enterocytes using Myo1A‐Gal4 (Jiang et al. 2009), each snoRNA increases longevity (Figure 2a). The expression of two (sno2 and sno3), or three (jouvence, sno2 and sno3) also similarly increases longevity, indicating that each snoRNA itself is sufficient to increase lifespan. Since Myo1A‐Gal4 is also transiently expressed during development (Morgan et al. 1994), to overcome the possibility that the observed longevity increases were due to developmental effects, we used two inducible drivers, as Mex‐GS and CG8997‐GS. Mex‐GS (Soulé et al. 2020) is expressed in several enterocytes, but not exclusively, as suggested by a transcriptomic analysis performed on the epithelium of the gut (Dutta et al. 2015). Nevertheless, the expression of each snoRNA individually with this driver only in adulthood increased lifespan, although the rescue is not as strong as with Myo1A for jouvence (Figure 2b–g). The RU486 itself does not have any effect on lifespan (Figure 2b), while the expression of the sno2&3 (Figure 2f), or the 3snoRNAs (Figure 2g) yielded a slightly different effect compared to Myo1A (Figure 2a), but nevertheless, still increases lifespan. Using the CG8997‐GS, which is expressed mainly in the R3 region of the gut, also leads to similar increases of lifespan (Figure S2a–e).

Since ISH, complemented by RT‐qPCR (Figure 1), revealed that the snoRNAs are also expressed in the fat body, we examined the effects of the rescue of their expression, individually and in combinations, using the fat body Lsp‐Gal4 driver. This resulted in increased lifespan to various degrees (Figure 2h). Interestingly, the sno3 was by far the most efficient in this experiment. Since the Lsp‐Gal4 is also known to be expressed during development (Benes et al. 1996), we also used the Lsp‐GS to express the snoRNA only in adulthood to circumvent any putative developmental effect. The expression of jouvence strongly increases lifespan; sno2 has a moderate effect, whereas sno3 has a negative effect, while the sno2&3 together present a moderate effect similar to sno2 alone (Figure S2f–j). These results suggest that only jouvence and the sno2 are active in the fat body in adults, and that the sno3 does not have an effect in the fat body in adulthood, but has a strong effect during development (Figure 2h).

### 2.3 Targeted Expression of Each snoRNA Prevents Neurodegenerative Lesions

**FIGURE 3:**
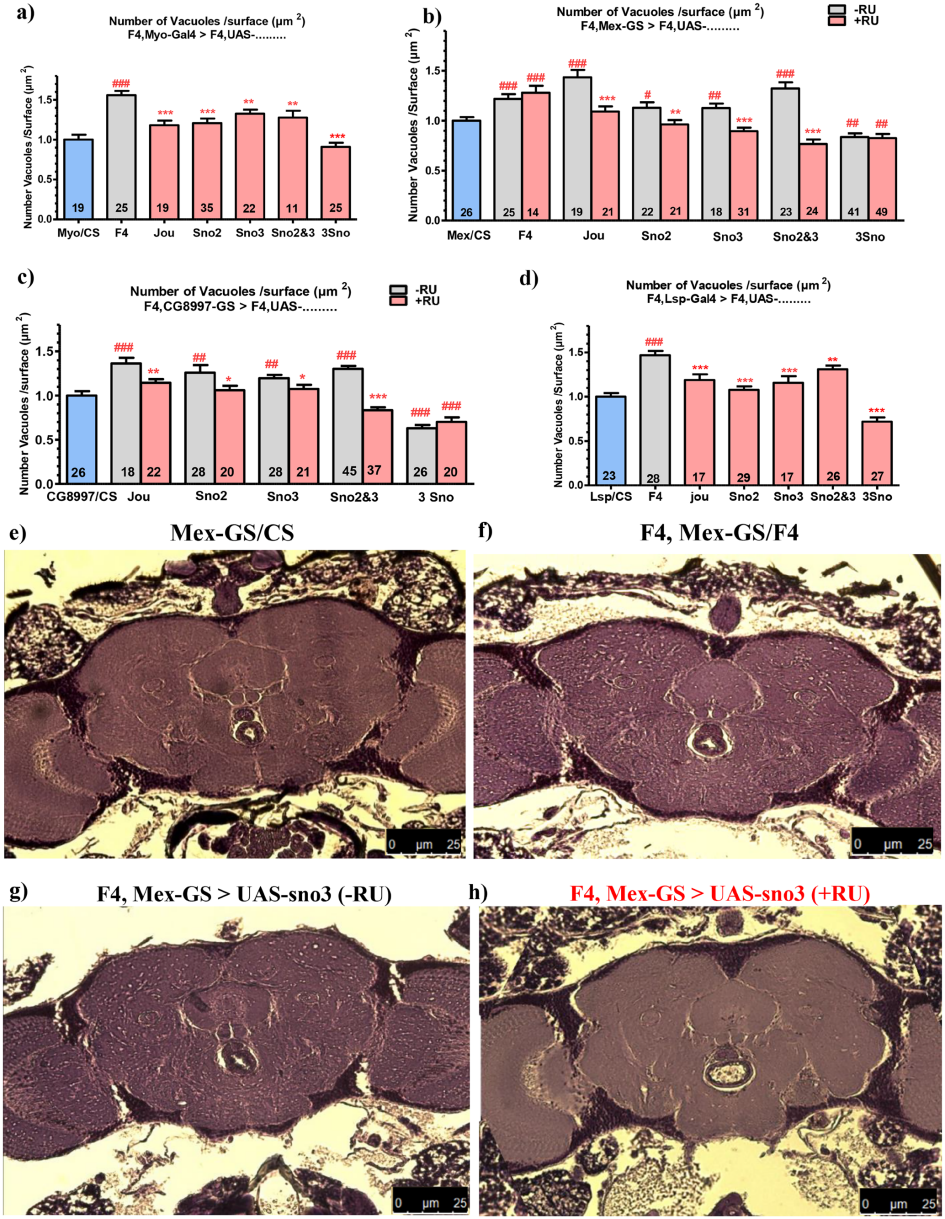
Quantification of vacuoles of neurodegenerative lesions in old flies. Each expression (rescue) was performed in the F4‐deleted genetic background. (a) Number of vacuoles for the Myo1A‐Gal4 driving each UAS‐snoRNA in the enterocytes. (b) Number of vacuoles for the Mex‐GS driving each UAS‐snoRNA in the enterocytes of F4‐deleted flies, without and with feeding RU486 to induce the expression of the UAS‐snoRNA transgenes. (c) similar, but using the driver line CG8997‐GS. (d) similar, but using the driver line Lsp2‐Gal4 to express the snoRNA in the fat body. Numbers within the histograms indicate the number of flies; error bars represent the mean ± SEM. *p*‐values were calculated using the student *t*‐test (Prism). Asterisks indicate significant differences compared to flies that never received RU (gray), while hashtags (#) highlight significant differences compared to wild‐type flies (Blue) (Myo/CS, Mex/CS, CG8997‐GS/CS, Lsp/CS) (*p < 0.05; **p < 0.005; ***p < 0.0005). (e–h) Micro‐photography showing the vacuoles in old flies of a Wild‐Type fly (e), in a F4‐deleted fly (f), in a F4, Mex‐GS>UAS‐sno3, without RU (g), and after induction with RU (h).

Since the re‐expression of each snoRNA in the F4‐deleted flies increases lifespan, we wondered if in parallel, the neurodegenerative lesions observed in old flies are also rescued or at least improved, indicating a putative neuroprotection. As we reported previously (Soulé and Martin 2020), the deletion of the 3 snoRNAs (F4) increases the number of neurodegenerative lesions (vacuoles) within the brain in old flies (Figure 3). Myo‐Gal4 driving the expression of jouvence within the enterocytes rescued the number of lesions (Figure 3a), as well as the total surface area of these lesions (Figure S3a). We observe similar effects with the sno2, sno3, sno2&3, and even a stronger effect with the 3snoRNAs (Figure 3a). However, with the sno2, sno3 or sno2&3, the surface area was not rescued, while it was significantly rescued with the 3 snoRNAs (Figure S3a). These results suggest that jouvence is the most efficient to rescue the neurodegenerative lesions, while when accompanied by sno2 and sno3 (3 snoRNAs altogether), the rescued effect is even stronger (both for the number of vacuoles and the surface area). Similar results are observed when each snoRNA are expressed only in adulthood, with Mex‐GS (Figure 3b) and CG8997‐GS (Figure 3c). Again, the strongest effect was produced with the 3 snoRNAs, although a slight improved effect is also observed without RU486 induction (see below for further explanation). For the surface area (Figure S3b,c), similar results are obtained with jouvence, while slight improvements are observed with sno2, sno3, sno2&3 or the 3 snoRNAs (see below for further explanation). Altogether, these results, obtained with three different drivers, indicate although with some differences, that the expression of the snoRNAs within the enterocytes is sufficient to rescue the neurodegenerative lesions in old flies. To test for the neuroprotective role of the three snoRNAs within the fat body, we expressed them, alone or in combinations, using the Lsp‐Gal4 driver. All of combinations partially rescued the F4 deletion phenotype by reducing the number (Figure 3d), and the surface area (Figure S3d) of the lesions. Again, the strongest effect was observed with the expression of the 3 snoRNAs. Thus, the expression of each snoRNA within the fat body also prevents the neurodegenerative lesions, suggesting that the gut defect could be compensated by a rescue in the fat body.

### 2.4 RT‐qPCR Confirms the Expression of Each snoRNA by P[Gal4] Lines

**FIGURE 4:**
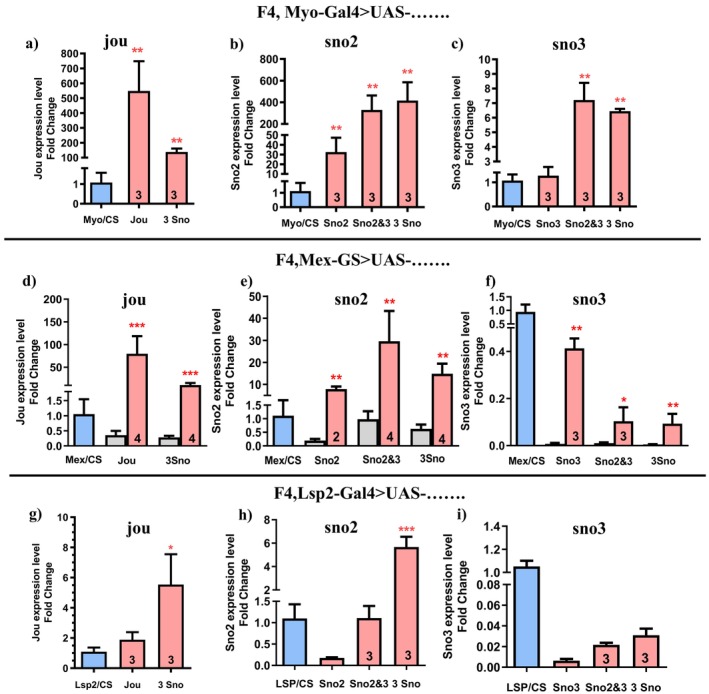
Expression level of each snoRNAs (RT‐qPCR) in rescued F4 flies. (a–c) RT‐qPCR (Taqman) results of jou (a), sno2 (b), or sno3 (c), following the expression of the appropriate snoRNA by the Myo1A‐Gal4 driver in the enterocytes (e.g., in a, the quantification of the sno2 and sno3 have not been performed since the flies are in F4 deleted genetic background, and thus they are absent, idem of the other genetic combinations). (d–f) idem for the Mex‐GS driver in the enterocytes, with (red/pink) and without (grey) RU486. (g–i) idem for the Lsp2‐Gal4 driver in the abdominal and pericerebral fat body. Statistics: For each snoRNA, comparison to control Myo/CS, Mex/CS and Lsp/CS used as reference (=1). (*p*‐values) (*p < 0.05; **p < 0.005; ***p < 0.0005). Errors bars represent the mean ± SEM (*p*‐value were calculated using the student *t*‐test, using Prism).

To validate that the snoRNAs are well expressed in the gut or in the fat bodies, we have performed RT‐qPCR. With Myo1A‐Gal4, jouvence is overexpressed 500 times higher, while it is 100 times higher with the 3 snoRNAs (Figure 4a). For the sno2, the overexpression is only 30 times higher, while it is 350 times higher with the sno2&3, and 400 times higher with the 3 snoRNAs (Figure 4b). For the sno3, the overexpression is exactly of one (perfect rescue level), while it is of 7 times higher with the sno2&3, and 6 times higher with the 3 snoRNAs (Figure 4c). Interestingly, we observe a strong difference in the expression level of the snoRNAs under the control of Myo1A‐Gal4, even though the UAS‐construct vector is the same for each snoRNA, and the transgenic insertion site within the genome is the same for the sno2, sno3, and sno2&3 (VK27, 89E11, chromosome 3L) thereby excluding any insertion site effect. Thus, this difference is likely not due to a difference in the transcription level, but rather due to the stability of the snoRNA itself, a question that remains to be addressed.

With the inducible Mex‐GS, we observed a similar situation, but with generally lower expression levels compared to Myo1A‐Gal4 (Figure 4d–f). For example, the level of jouvence is 100 fold compared to 400 for Myo1A‐Gal4, and similarly for sno2 (Figure 4e) and sno3 (Figure 4f). However, the RT‐qPCR quantification has revealed that in any of the Mex‐GS>UAS‐snoRNA, we detect a slight level of snoRNA (Figure 4d–f, in gray), without feeding the flies with RU486, suggesting a weak leakage of uninduced snoRNA expression. Whether this expression leak is due to the Gene‐Switch that is not well locked, a phenomenon already reported for several Gene‐Switch lines (Poirier et al. 2008), or due to the leakage of UAS‐snoRNA itself, remains to be determined. The variation in the overexpression level of snoRNAs likely contributes to the differences observed in the degree of rescue of longevity and/or neurodegenerative lesions. In other words, even though quantitatively the phenotypes might be affected differently by different levels of snoRNAs, the actual rescue is not in question.

Under the control of the Lsp2‐Gal4 driver, the level of jouvence is doubled compared to control flies (Figure 4g), while it is increased 6‐fold for the 3 snoRNA. For the sno2, the level is only 0.1 compared to control (Figure 4h), while it is equal to the control for the sno2&3 and 6‐fold for the 3 snoRNAs. For the sno3, as for the sno2, it is only 0.1 compared to control (Figure 4i), while it is 0.2 for the sno2&3 and 0.3 for the 3 snoRNA. Here, although this quantification has been done on whole flies, obviously the expression level is much lower than with the other gut Gal4 drivers. Moreover, the expression levels of jouvence, sno2, and sno3 are clearly very different, which again could contribute to the differences observed in the longevity and brain lesion phenotypes.

### 2.5 Targeted Expression of Each snoRNA Rescues Triglycerides and Sterol Levels

**FIGURE 5:**
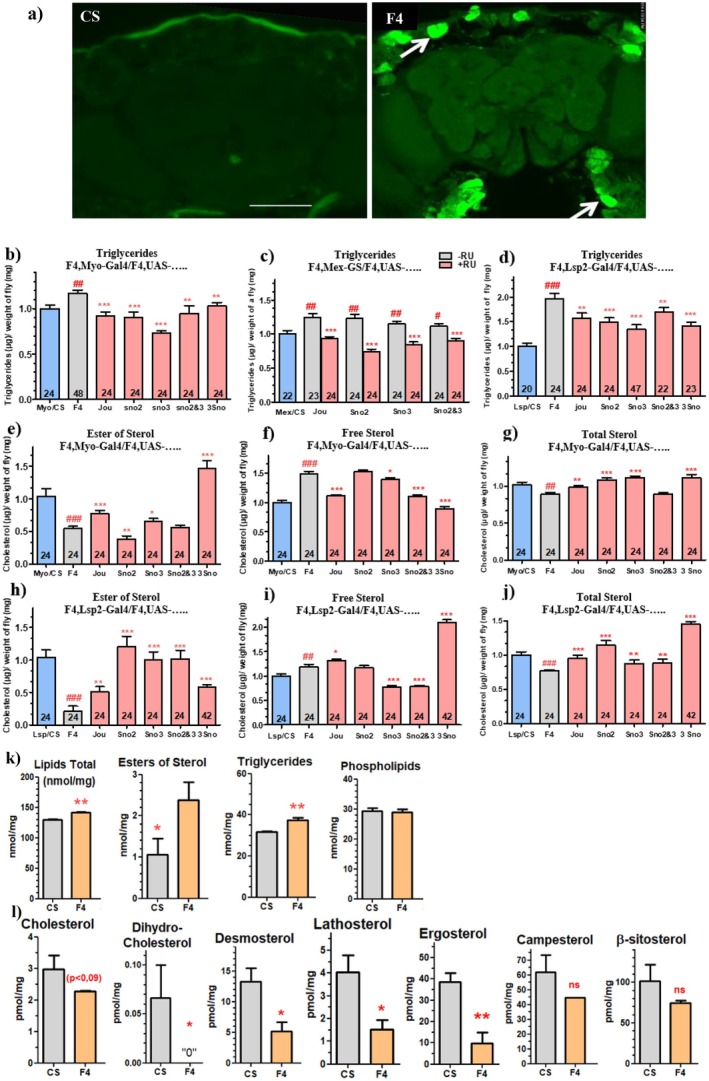
Deregulated Triglycerides and Sterols are rescued by targetted expression of snoRNA. (a) Immuno‐Histological stainings using anti‐caspase‐3 antibody on 40 day‐old flies reveal aggregates (white arrows) located in the pericerebral fat body (F4‐deleted flies) compared to Wild‐Type (CS) control flies (scale bar = 100 μm). (b) TG dosage on whole young flies (7 day‐old), following the expression of each snoRNA using Myo1A‐Gal4 line. (c) Dosage of TG using Mex‐GS, with and without feeding RU486. (d) Dosage of TG following the expression of each snoRNA in the abdominal and pericerebral fat body, using Lsp2‐Gal4. Quantification of sterol‐ester (e), free (f), and total (g) levels in wild‐type controls (CS), F4‐deleted flies (F4), and flies expressing each snoRNA in the enterocytes of the gut under the control of Myo1A‐Gal4. Quantification of sterol‐ester (h), free (i), and total (j) levels in flies expressing each snoRNA in the fat body under the control of Lsp2‐Gal4. Numbers in the histograms indicate the number of flies; error bars represent the mean ± SEM (*p < 0.05; **p < 0.005; ***p < 0.0005). *p*‐values were calculated using the student *t*‐test (Prism). Asterisks indicate significant differences compared to flies that never received RU (gray), while hashtags (#) highlight significant differences compared to wild‐type flies (Blue) (Myo/CS, Mex/CS, CG8997‐GS/CS, Lsp/CS). (k) Lipidomics analysis reveals the precise amount of each class of lipids. (l) Amounts of different classes of sterol. All of them are decreased, although the campesterol and β‐sisterol are not statistically different. Remarks that the Y‐scales are different for each sterol, and notably that the phytosterol (ergosterol, campesterol and β‐sisterol) are much higher.

To characterize the neurodegenerative lesions within the brain, and specifically to determine if these lesions could be due to apoptosis, we have performed immuno‐histological staining with an anti‐caspase‐3 antibody, which reveals several aggregates and a hypertrophy of the pericerebral fat body (Figure 5a) in F4‐deleted flies, without any striking staining within the brain. To investigate this further, we have quantified, in the whole fly, the triglyceride (TG) levels. First, in F4‐deleted flies with Myo1A‐Gal4, the level of TG is increased (Figure 5b). The expression of jouvence in enterocytes rescues the level of TG. The expression of sno2, or sno3, or sno2&3, or the 3 snoRNAs leads to similar results, with a maximum effect observed with the sno3. Similar effects were obtained when the snoRNAs are expressed only in adulthood using Mex‐GS after RU486 feeding (Figure 5c). The expression of each snoRNA in the fat body using the Lsp2‐Gal4 (Figure 5d) also rescues (decreases) the level of TG, again, with a maximum effect observed with the sno3.

We next asked if the sterols, another major components of the metabolism, were also deregulated. We quantified the sterols on the whole flies, as for the TG. The ester of sterol is strikingly reduced (Figure 5e), and the free sterol is increased (Figure 5f), while the sum of both, the total‐sterol, is just slightly decreased (Figure 5g) in F4‐deleted flies compared to wild‐type control. The expression of jouvence in the enterocytes (Myo1A‐Gal4) rescues the sterol‐ester and the free sterol phenotypes (Figure 5e), while the expression of the sno2 does not have any effect on sterol levels (Figure 5f). The expression of sno3 produced only a mild rescue effect (Figure 5e–g), whereas the expression of sno2&3 did not have any effect on sterol‐ester, but did on free sterol (Figure 5g). Finally, the expression of the 3 snoRNAs rescues the sterol‐ester and the free‐sterol (Figure 5e,f). In the fat body, the expression of sno2, sno3, or sno2&3, rescues the sterol‐ester, while the free sterol is rescued only by sno3 or sno2&3 (Figure 5h,i). For jouvence and the 3 snoRNAs, the ester of sterol is partially rescued with any rescue effect on free sterol (Figure 5h,i). In conclusion, strikingly, the main effects on sterol (both‐ester and ‐free) are due to the presence of jou in the gut, either when it is expressed alone or when it is included in the cluster of the 3 snoRNAs. However, in the fat body, the primary effect on sterol (both esterified and free) results from sno3, either alone or when co‐expressed with sno2.

### 2.6 A Lipidomic Analysis Reveals Different Classes of Lipids and Sterols

To investigate further the nature of the deregulated lipids and sterols, as well as their fatty‐acid components, we performed a lipidomic analysis. In F4‐deleted flies, we observe an increase in total lipids, triglycerides, and ester of sterols, while the amount of phospholipids is not modified (Figure 5k). Conversely, the different forms of sterol, as the cholesterol, dihydro‐cholesterol, desmosterol, lathosterol, and ergosterol are substantially decreased, while the campesterol and the β‐sitosterol do not differ statistically (Figure 5l) (note that the levels/quantity of phytosterols as ergosterol, campesterol and β‐sitosterol are much higher).

In contrast, the precise determination of the fatty‐acid (FA) components is more complex. The results indicate that the deletion of the 3 snoRNAs (F4‐deletion) perturbs quantitatively some specific FA, especially the short ones (12:0 and 14:0). Indeed, overall, for the total lipids and triglycerides, the short FA, such as 12:0 and 14:0, are increased, while the longer ones (> 16:0) are decreased, with some exceptions (Figures S4 and S5). The situation for sterol‐ester is quite similar, except that the 16:0 are increased (Figure S6). Finally, for phospholipids, there is no striking modification except a decrease in 14:1 (Figure S7).

### 2.7 Neurodegenerative Lesions Are due to Peroxidized Lipids and Sterol Deregulation

**FIGURE 6:**
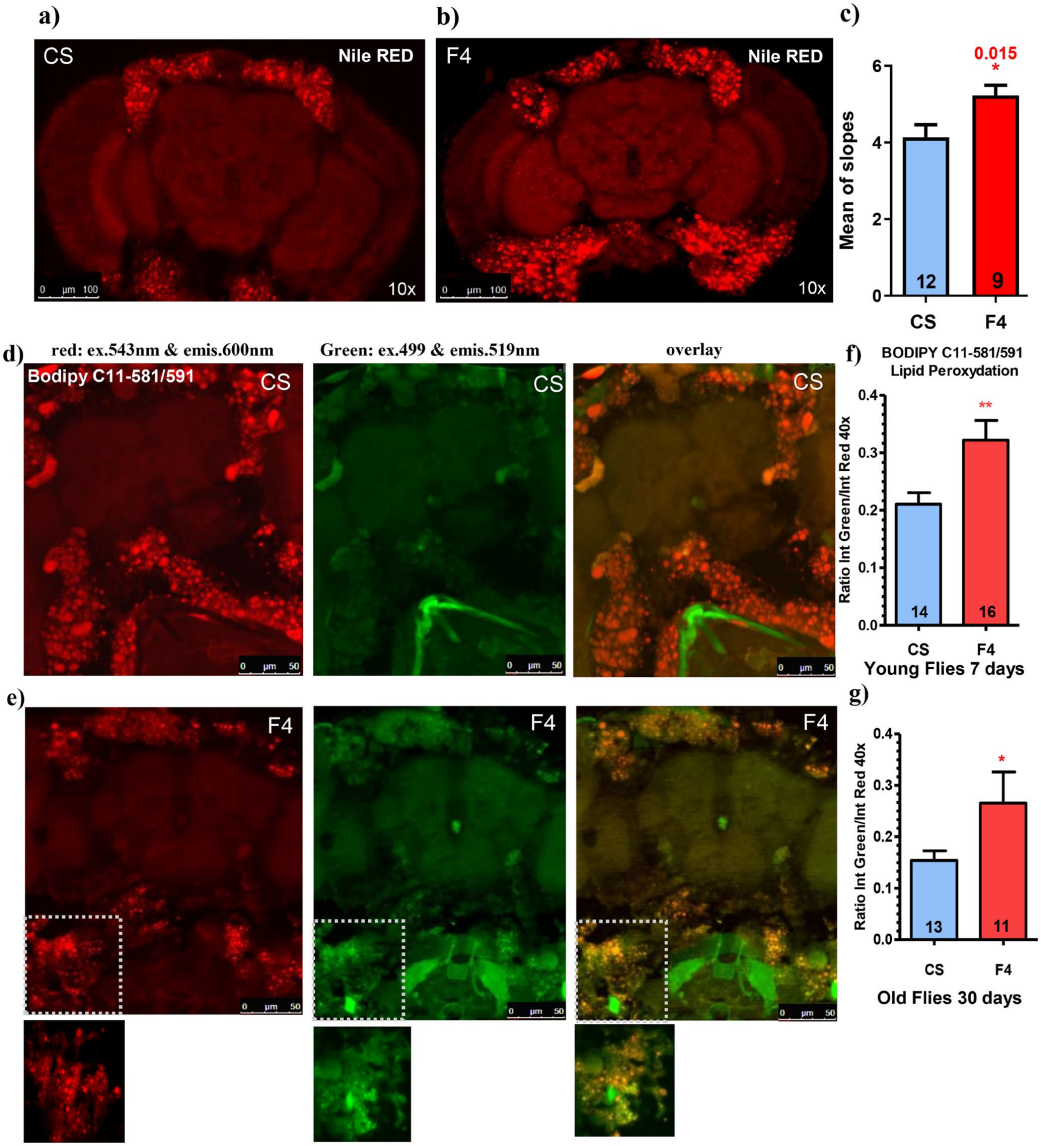
Nile Red and BODIPY C11‐581/591 highlight the nature of the neurodegenerative lesions. (a, b) Nile Red staining of the brain shows the pericerebral fat body in Wild‐Type CS (a) and F4‐deleted flies (b). Several unique puncta (or aggregates) are visible, principally in F4 flies (b). The double nature of the Nile Red, a shift of emission from red to yellow dependent on the degree of hydrophobicity of lipids, allows one to discriminate the nature of the lipids. In (c) the mean of the slope of the regression line of the Nile red/yellow emission ratios indicates a higher amount of free‐cholesterol in the aggregates. Numbers in the histograms indicate the number of flies; error bars represent the mean ± SEM. Asterisks indicate significant differences. (*p < 0.05). *p*‐value were calculated using the student *t*‐test (Prism). (d–g) BODIPY C11‐581/591 staining of the brain shows the pericerebral fat body in Wild‐Type CS (d) and F4‐deleted flies (e). First column, in red: Excitation at 543 nm & emission at 600–650 nm. Second column, green: Excitation at 499 nm & emission at 519 nm. Third column, overlay. The quantification of the green/red ratio confirms that F4 flies have more peroxidized lipids than control flies, both in young (f) and old flies (g). Numbers in the histograms indicate the number of flies; error bars represent the mean ± SEM. Asterisks indicate significant differences (*p < 0.05; **p < 0.005). P‐values were calculated using the student *t*‐test (Prism).

Given the deregulated general metabolic parameters (TG and sterols) in F4 flies, we investigated the nature of the brain lesions by performing different histological stains on the brain. Lipid staining using Nile Red reveals several positive puncta (or aggregates) spread all over the brain in F4‐deleted flies compared to controls (Figure 6a,b), resembling the vacuoles observed in the paraffin section of the old flies, ascribed to neurodegenerative lesions (Figure 3). To determine if these points/aggregates are associated with glial cells or neuronal cells, similar stains were performed using repo‐Gal4 > UAS‐GFP to label the glial cells, and the n‐syb‐Gal4 > UAS‐GFP to label the neurons. Double‐stainings reveal that the Nile Red overlaps with the neuronal tissue but not with the glial cells (Figure S8). Nile Red is a fluorescent lipophilic dye characterized by a shift of emission from red to yellow corresponding to the degree of hydrophobicity of lipids [34]. More specifically, polar lipids such as phospholipids are stained in red, while neutral lipids, such as triglycerides and cholesterol esters, which are present in lipid droplets, are stained in yellow. Thus, we took advantage of this unique property to quantify the ratio of red and yellow emission in order to discriminate the labeled lipids. According to a lipid reference (Figure 1 in Diaz et al. 2008), the Nile Red aggregates in F4‐deleted flies correspond to an increase of free sterol at the expense of sterol ester (Figure 6a–c).

Since lipids could be peroxidized, we also measured the lipid peroxidation using the BODYPY C11‐581/591, which reveals the non‐peroxidized lipids in red, while the peroxidized ones are in green (Bailey et al. 2015). Figure 6d shows that the controls have just a little green stain, whereas F4 (Figure 6e) has a more intense green stain. The quantification of the green/red ratio reveals that the F4‐deleted flies have more peroxidized lipids than controls, both in young and old flies (Figure 6f,g), specifically in the pericerebral fat body.

### 2.8 Targeted Expression of Each snoRNA Within Enterocytes Rescues the Expression Levels of Genes Involved in Lipid Metabolism to Different Extents

**FIGURE 7:**
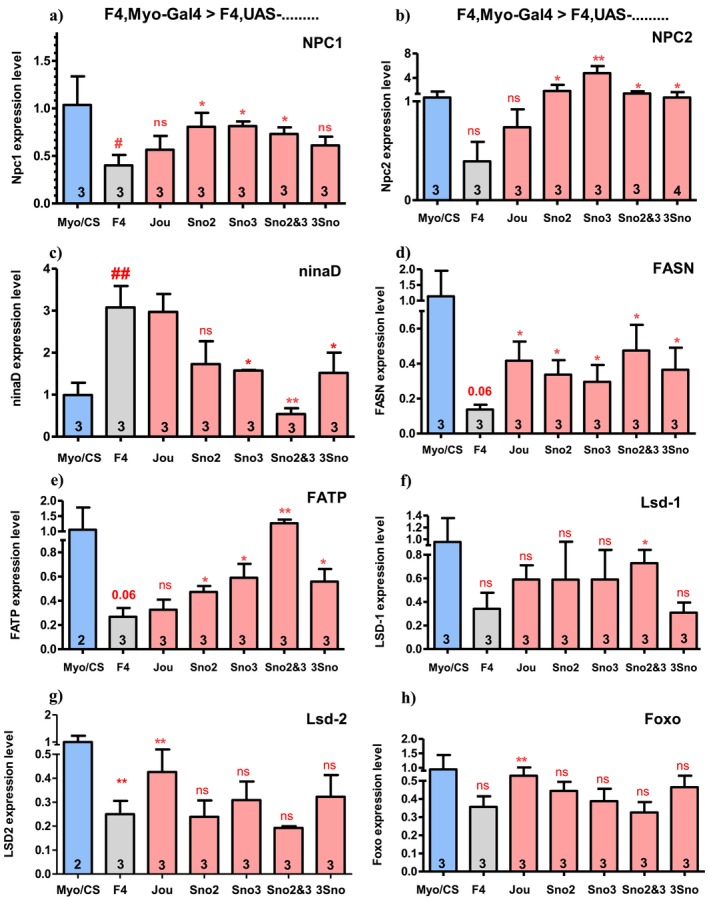
Different genes are differentially rescued by the snoRNA (RT‐qPCR). RT‐qPCR (SybGreen) of 8 different encoding genes performed on dissected gut of Controls, F4‐deleted flies and targeted expression of each snoRNA in the enterocytes driven by Myo1A‐Gal4. (a) NPC1. (b) NPC2. (c) ninaD. (d) FASN. (e) FATP. (f) Lsd1 (perilipin‐1). (g) Lsd2 (perilipin‐2). (h) FOXO. Statistics: The expression level of each gene in Control Wild‐Type (Myo1A/CS) is used as reference (fixed to 1). Three independent biological replicates (*n* = 3). (*p*‐values) (*p < 0.05; **p < 0.005; ***p < 0.0005). Errors bars represent the mean ± SEM. (*p*‐value were calculated using the student *t*‐test, using Prism).

To decipher the molecular mechanisms linking the epithelium of the gut, the metabolic parameters, and the neurodegenerative lesions, we quantified by RT‐qPCR a selection of genes involved in the metabolism of lipids and sterols. Based on the previous transcriptomic analysis performed on the dissected gut (Soulé et al. 2020), first, we analyzed NPC1, NPC2, and ninaD, three genes involved in the sterol (cholesterol) metabolism. NPC1, decreased in F4, is partly rescued by the expression of sno2, sno3, and sno2&3, but neither by the 3 snoRNAs nor by jouvence (Figure 7a). NPC2, also decreased in F4, is well rescued by the re‐expression of sno2, sno3, sno2&3, and the 3 snoRNAs, but not by jouvence (Figure 7b). ninaD, which in contrast is increased in F4 (Figure 7c) (Soulé et al. 2020; Soulé and Martin 2020), is rescued only by the re‐expression of sno3, or sno2&3, or the 3 snoRNAs. Altogether, these results indicate that the rescue of the mRNA levels of NPC1 or NPC2 depends on sno2 and/or sno3, but not jouvence, while ninaD is achieved solely by sno3, since the presence of sno3 is mandatory to rescue it (in sno3, sno2&3, or 3 snoRNAs).

We also investigated some genes involved in the fatty‐acid and triglycerides metabolism. The gene FASN (fatty‐acid synthase), decreased in F4 (Soulé et al. 2020), is partly rescued by the re‐expression of any snoRNA (jouvence, sno2, sno3, sno2&3, and 3 snoRNAs) (Figure 7d). FATP (fatty‐acid transporter protein), also decreased in F4, is not rescued by jou, but is partly rescued by the re‐expression of the sno2, sno3, and the 3 snoRNAs, while it is perfectly rescued by the expression of the sno2&3 (Figure 7e). We also checked the expression level of the two perilipin genes (Lsd1 and Lsd2), which are known to be crucial for the formation of lipid droplets (Beller et al. 2010; Bi et al. 2012). While both are decreased in F4 (Figure 7f,g), only Lsd1 is rescued by the sno2&3, while Lsd2 is rescued only by jouvence. Unexpectedly, for the two perilipins, the lack of rescue following the re‐expression of each snoRNA in the gut seems to be, at least partly, due to a larger variability observed between the three independent replicates.

We also quantified the gene FOXO, one of the crucial genes of the insulin‐signaling pathway (Bolukbasi et al. 2017). FOXO is decreased in F4, confirming the RNA‐Seq (Soulé et al. 2020), while it is only rescued by the re‐expression of jouvence (Figure 7h). Interestingly, this rescue pattern is a mirror image of the rescue pattern observed for the ninaD gene. Similar results obtained by RT‐qPCR on the same set of genes were observed with the Mex‐GS line after RU486 feeding, indicating that the expression of these genes within the epithelium of the gut only in adulthood is sufficient for the rescues (Figure S9). In addition, the same genes have been investigated using the Lsp2‐Gal4 expressed in the fat body (Figure S10). Briefly, NPC1 and NPC2 are not rescued, while the expression of jouvence rescues the ninaD level, almost as a mirror image of the effects of the snoRNAs in the gut. For the FASN and FATP, the rescues in the fat body are similar to the rescues in the gut (although with some slight differences). Lsd2, like in the gut, is not rescued, except by the 3 snoRNAs. Finally, for FOXO, the results present some variability, due to a large difference between the replicates.

## 3 Discussion

We have reported that the deletion of the entire cluster of 3 snoRNAs including jouvence (in the F4‐deletion) decreases the longevity of flies, while their re‐expression, individually or in different combinations, specifically within the enterocytes of the gut of the F4 deletion flies rescues the longevity phenotype to various degree. This was demonstrated with three independent driver lines: Myo1A‐Gal4, Mex‐GS, and CG8997‐GS. Although the expression patterns of the Mex‐GS, and CG8997‐GS do not completely cover the entire midgut (in contrast to Myo1A‐Gal4) the longevity is nevertheless improved, indicating that the re‐expression of the snoRNA only in adulthood is sufficient to rescue the phenotype. Moreover, as revealed by the ISH and corroborated by RT‐qPCR (Figure 1), the 3 snoRNAs are also expressed in some cells of the abdominal and pericerebral fat bodies. Surprisingly, the re‐expression of each snoRNA individually, or in different combinations, in the fat body using the Lsp‐Gal4 (Figure 2h), or adulthood‐specific Lsp‐GS (Figure S2) also improves lifespan (except for the sno3 which is the inverse‐deleterious: Figure S2i). However, also here, the expression of the sno3 leads, to various degree, to much better longevity than jouvence, and even than the 3 snoRNAs (Figure 2h). This discrepancy between the Lsp‐Gal4 > sno3 (Figure 2h) and the Lsp‐GS induced sno3 expression only in the adulthood (Figure S2i) suggests that the sno3 might have a significant function during development.

Since the snoRNA overexpressing flies live longer than F4‐deleted controls, we investigated if these longer‐living flies have more or fewer brain defects. F4‐deleted old flies present more neurodegenerative lesions (vacuoles) than the wild‐type controls (which also present some lesions), as previously reported (Soulé and Martin 2020). However, the re‐expression of each snoRNA (individually or in combinations) within the gut rescues both the number (Figure 3) and the surface area of neurodegenerative lesions (Figure S3). As was the case for the longevity, these effects are obtained with 3 independent gut drivers, two of which were expressed only in adulthood, indicating that the expression of the snoRNA is sufficient only in adulthood. Moreover, the expression of each snoRNA within the fat body (Lsp‐Gal4) is also sufficient to improve the neurodegenerative lesions (both the number and the surface area) with a better effect when we express the 3 snoRNAs together. Therefore, these results indicate that the defects induced by the missing snoRNA within the enterocytes could be compensated by the re‐expression of them in the fat body. They also reveal a correlation (though it is not a perfect linear correlation) between the longevity of the flies and the severity of the brain lesions.

Since none of the snoRNAs are expressed within the brain, to trace a causal relationship between the tissues that express the snoRNA (the gut and to lesser extent, the fat body) and the brain lesions, we quantified a set of metabolic parameters. These experiments were firstly guided by the fact that immuno‐histological stainings of the heads and brains have revealed that the main visible histological lesions were localized in the pericerebral fat body, indicating notably a hypertrophy of the fat body (Figure 5). Thus, while the TG are increased in F4, a normal level of TG is rescued to various extents by the different snoRNAs when they are expressed alone or in different combinations within the enterocytes (Figure 5). Similar effects (although with some variability) are also obtained with the expression of the snoRNAs within the fat body. For the sterols and their various forms, the sterol‐esters seem to be the most affected (a decrease) in F4, while the free sterols are increased, for a total sterol level being only slightly affected (Figure 5). However, in contrast to the TG, which were rescued by all the snoRNAs, the sterol and notably the sterol‐ester levels are rescued solely by jouvence (either alone or included within the 3 snoRNAs). Interestingly, these last results also correlate to the longevity and the neurodegenerative lesions. Therefore, they allow us to suggest that the metabolic parameters (TG and sterols) could fulfill the gap between the gut (or the fat body) and the neurodegenerative lesions. In other words, the deregulation of the metabolic parameters due to the missing snoRNAs, primarily within the gut and secondarily within the fat body, chronically leads to neurodegenerative lesions.

To characterize the nature of the brain lesions, different stainings have been performed. First, the Nile Red, known to stain the neutral lipids (Diaz et al. 2008), has revealed several small aggregates within the neuronal tissues, as confirmed by the double staining with n‐Syb‐Gal4 (Figure S8). Moreover, the Nile Red fluoresces in different wavelengths, from red to yellow in relation to the degree of hydrophobicity of lipids. This last property has suggested that these aggregates are enriched in free sterols. Second, the BODIPY C11‐581/591, which fluoresces differentially according to the degree of peroxidation of lipids, has revealed a higher degree of lipid peroxidation in F4, especially in the pericerebral fat body. Altogether, these various stains, either in the fat body or in the brain, in conjunction with the deregulation of some metabolic parameters (TG and sterols), suggest that the neurodegenerative lesions might be a consequence of the chronic deregulation of the metabolic parameters induced by the missing expression of the different snoRNAs, occurring in the gut and/or the fat body.

To strengthen this causality focussing on the relationship between the snoRNA within the enterocytes and the deregulated metabolic parameters, we have quantified the expression of several genes involved in the lipid and sterol metabolism from the dissected gut of various snoRNA rescued genotypes. In agreement to the previous transcriptomic analysis (Soulé et al. 2020), we have confirmed that the re‐expression of all snoRNAs, except for jouvence, is sufficient to increase (partly rescue) the level of the NPC2a gene, one of the main genes regulating cholesterol homeostasis (Huang et al. 2007), and the NPC1a, a gene involved in intracellular sterol trafficking (Phillips et al. 2008). However, ninaD is not rescued by jouvence, but instead seems to require the presence of the sno3, since it is rescued by sno3 alone, or sno2&3 or by the 3 snoRNAs. Altogether, these results observed with three main genes involved in sterol homeostasis leading to chronic sterol metabolic defects likely participate in neurodegeneration. FASN and FATP, two genes involved in the fatty‐acid and triglycerides synthesis and homeostasis (Heier and Kühnlein 2018), are also rescued by all the snoRNAs, except that FATP is not rescued by jouvence. However, it is not the same for all analyzed genes. For example, perilipin Lsd1 is rescued by only the sno2&3, and Lsd2 is not rescued by any snoRNA. These differences in the rescue of different genes by the re‐expression of the different snoRNA indicates a rather complex regulatory mechanism controlled by the snoRNAs within the epithelium of the gut.

Considered together, at the organismal level, our results demonstrate a tight relationship between the expression of the snoRNA within the enterocytes controlling the expression of several genes, which allows a precise regulation of several metabolic parameters. Moreover, these results suggest that the defects induced by the lack of the 3 snoRNAs within the gut could be compensated by the re‐expression of them in the fat body, a result that reinforces the fact that the neurodegenerative lesions as well as longevity are consequences of the deregulation of the metabolic parameters, such as TG and sterol. Since the brain continuously needs precise regulation of various nutrients to maintain its homeostasis throughout the organism's life, the deregulation of one of several of them, particularly chronically, such as TG and sterol, leads to the development of age‐progressive neurodegenerative lesions. In brief, we have shown that the brain lesions observed in aged flies can be a consequence of the perturbation of the gut homeostasis, indicating that a gut‐brain axis is involved in maintaining a healthy brain.

## 4 Materials and Methods

### 4.1 *Drosophila* Lines

*Drosophila melanogaster* flies were grown on standard medium (1.04% agar, 8% cornmeal, 8% brewer yeast and 2% nipagin as a mold inhibitor) at 25°C, 12:12 light:dark cycle in a humidity‐controlled incubator. For aging experiments, 15 adult female flies were crossed with 10 males per vial, and were transferred to new food vials every 2 days. Wild‐Type *Canton‐S* (CS) flies were used as control. The results described in this study were obtained from females. All genotypes were outcrossed to the wild‐type CS (cantonization) at least 6 times to homogenize the genetic background. RU486 (Mifepristone) (*Sigma‐Aldrich*, Cat. #M8046) to induce the Gene‐Switch activity was dissolved in ethanol and mixed into the media when preparing food vials. RU486 doses used were 25 μg mL^−1^ final concentration. Myo1A‐Gal4 was kindly provided by B.A. Edgar (Heidelberg, Germany), CG8997‐GS from H. Tricoire (Paris, France). Mex‐GS (in VK02 site), UAS‐jou (inserted randomly on the third chromosome), UAS‐sno2 (in VK27 site), UAS‐sno3 (in VK27 site), UAS‐sno2&3 (in VK27 site), and UAS‐3snoRNAs (in attP2 site), have been generated in our laboratory (Soulé et al. 2020). These various transgenic lines were then introduced into the F4‐deletion genetic background by standard genetic crosses.

### 4.2 Lifespan Analysis

Following amplification, flies were harvested every day after hatching. Female flies were maintained with males in fresh food vials for 4 days at a density of 25 individuals per vial. On Day 4, males were removed, while females were placed in a cage (about 200 to 300 females per cage), as in Soulé et al. (2020). The aging animals were transferred to fresh food every 2 days, and the number of dead flies was scored. The lifespan plots were generated by calculating the percentage of survivorship every 2 days and plotting viability as a function of time (days) using the log‐rank test (Yang et al. 2011).

### 4.3 Quantitative RT‐qPCR

Expression level of each snoRNA was measured on dissected gut or whole flies from 7‐days‐old female flies, as in Soulé et al. (2020). Quantitative RT‐qPCR was performed on a QuantStudio‐3 instrument (Applied Biosystem/ThermoFisher). For the snoRNA, we use a Taqman probe for each snoRNA (ThermoFisher). All assays were done in triplicate. Data were analyzed according to the ΔΔCt method, and normalized to RP49 levels. For the encoding genes, primers used are summarized in the Table S1.

### 4.4 Brain Histology

- Paraffin Section

Flies were filled in collars and fixed for 4 h in fresh Carnoy's fixative (ethanol: chloroform: acetic acid at 30:15:5) as in (Soulé and Martin 2020). Quantification of neurodegenerative lesions was performed on the entire brains using ImageJ software. Data are expressed as number of vacuolar/lesions per μm^2^ of measured surface.

b Nile Red and BODIPY C11‐581/591 Labelling

All histological experiments were carried out on pre‐fixed cryostat sections of the head from adult females aged of 7 days. For the Nile‐Red, a stock solution at 10% (Sigma‐Aldrich, Cat. #72485) is diluted in DMSO. After two washes of PBS‐Tween 0.05% (PBST), the head sections are incubated for 30 min in the dark, with the Nile Red solution diluted 1/10 from an intermediate solution at 0.05% freshly prepared in PBST. The head sections are washed 2X with PBST, and mounted on glass slide in Mowiol. The brain are observed with a fluorescent microscope (Leica DM 600B) at two excitations wavelength of 499 nm and 543 nm, and at an emission of 519 and 600–650 nm, respectively. The photos are acquired with a digital camera (Hamamatsu C10600 ORCA‐R2).

For the BODIPY C11‐581/591 labelling, in contrast to other histological staining, the heads of the flies are not fixed. The head sections are performed directly on frozen heads, washed 2× with PBS, and incubated with the BODIPY C11‐581/591 solution (D3861, ThermoFisher) diluted at 1/500 from a stock solution at 2 μM, during 30 min. The head sections are washed 2X with PBST, mounted on glass slides in Mowiol, and observed in a microscope using two different excitation wavelengths, at 499 and 543 nm, and an emission at 519 and 600–650 nm, respectively. To calculate the ratio of peroxidized versus non‐peroxidized lipids, ImageJ (FIJI) is used. The intensity of the red and the green fluorescence is measured on the same image, and the ratio IntDen‐Green/IntDen‐Red is calculated for each measurement.

### 4.5 Triglycerides Measurement

The Triglycerides (TG) quantification has been performed with a colorimetric method using the enzymatic kit “LiquiColor TG kit” (Stanbio, Cat. #2200‐430), as described in Soulé and Martin (2020). The TG determination is performed on 24 individual 7 day‐old female flies.

### 4.6 Sterol Measurement

Sterols (including cholesterol) in flies were measured as described in Soulé and Martin (2020). Measurements were carried out on 24 single 7‐day‐old adult female flies. Free sterol and sterol‐ester levels were measured using the Amplex Red cholesterol assay kit (*Invitrogen*, Cat. #A12216), according to the manufacturer's instructions. Notice that this enzymatic‐based assay (kit) also measures all other sterols, and not solely cholesterol (Serrano et al. 2024).

### 4.7 In Situ Hybridization (ISH)

For whole flies ISH, we used 5 day‐old females, exactly as described in Soulé et al. (2020).

### 4.8 Analysis of Expression Pattern of P[Gal4] and P[Gene‐Switch] Lines

All histological experiments were carried out on dissected guts from adult females aged 7 days. To determine the expression pattern of Gal4 lines, samples were fixed in 4% PFA for 15 min, washed three times with PBS, and mounted in Mowiol. Images were collected using a Leica DM 600B light microscope (*Leica*, Germany), equipped with a Hamamatsu C10600 ORCA‐R^2^ digital camera.

### 4.9 Lipidomic Analysis

Lipid analysis was performed on the Functional Lipidomics Platform acknowledged by IBiSA (Infrastructure in Biology, Health and Agronomy) at ENSA, Lyon, exactly as described in Jaque‐Cabrera et al. (2025).

### 4.10 Statistical Analyses

Statistical comparisons were done with GraphPad Prism. Data were analyzed using the student *t*‐test. Significance levels in figures were represented as *p < 0.05, **p < 0.01, ***p < 0.001. All quantitative data are reported as the mean ± SEM (standard error of the mean). Lifespan assays were subjected to survival analysis (log‐rank test) using the freely available OASIS software (Yang et al. 2011). Table S2 for the detailed statistics.

## Author Contributions

Jean‐René Martin conceived and designed the experiments. Sara Al Issa, Théo Gauvrit, Patricia Daira, and Nathalie Bernoud‐Hubac performed experiments and analyzed data. Jean‐René Martin wrote the manuscript with input from all authors.

## Funding

This work was supported by Agence Nationale de la Recherche, Aging‐jou.

## Conflicts of Interest

The authors declare no conflicts of interest.

## Supporting information


**Figure S1:** Expression Pattern of drivers CG8997‐GS, Mex‐GS, Lsp‐GS and Lsp2 Gal4, in F4‐Deleted flies.
**Figure S2:** Targeted expression of each snoRNA in the enterocytes or in the fat body increases lifespan.
**Figure S3:** Quantification of Surface Area of neurodegenerative lesions in old flies.
**Figure S4:** Fatty‐Acid (FA) composition of Total Lipids. The short FA (12:0 and 14:0) are increased, while the long FA (> 16:0) (except 20:0), are decreased.
**Figure S5:** Fatty‐Acid (FA) composition of Triglycerides. The short saturated FA (12:0 and 14:0) are increased, while some monounsaturated or long FA (14:1, 16:1n‐7, and > 16:0) are decreased.
**Figure S6:** Fatty‐Acid (FA) composition of Ester of Sterols. Several FA are decreased, while only the 16:0 is increased.
**Figure S7:** Fatty‐Acid (FA) composition of Phospholipids. The majority of FA are not modified.
**Figure S8:** The Nile‐Red overlaps with the neuronal tissue but not with the glial cells in old flies.
**Figure S9:** Different encoding genes are differentially rescued by the snoRNA driven by Mex‐GS (RT‐qPCR).
**Figure S10:** Different encoding genes are differentially rescued by the snoRNA driven by Lsp‐Gal4 (RT‐qPCR).
**Figure S11:** The primary sequences of the snoRNA‐2 and snoRNA‐3 present a high degree of identity.

## Data Availability

The authors declare that all the data and the methods used in this study are available within this article. Supporting Information is available from the corresponding authors upon reasonable request.
